# Prediction of Four Kinds of Simple Supersecondary Structures in Protein by Using Chemical Shifts

**DOI:** 10.1155/2014/978503

**Published:** 2014-06-18

**Authors:** Feng Yonge

**Affiliations:** College of Science, Inner Mongolia Agriculture University, Hohhot 010018, China

## Abstract

Knowledge of supersecondary structures can provide important information about its spatial structure of protein. Some approaches have been developed for the prediction of protein supersecondary structure. However, the feature used by these approaches is primarily based on amino acid sequences. In this study, a novel model is presented to predict protein supersecondary structure by use of chemical shifts (CSs) information derived from nuclear magnetic resonance (NMR) spectroscopy. Using these CSs as inputs of the method of quadratic discriminant analysis (QD), we achieve the overall prediction accuracy of 77.3%, which is competitive with the same method for predicting supersecondary structures from amino acid compositions in threefold cross-validation. Moreover, our finding suggests that the combined use of different chemical shifts will influence the accuracy of prediction.

## 1. Introduction

The prediction of protein structure is always one of the most important research topics in the field of bioinformatics. However, it is very difficult to predict the spatial structure directly from the protein sequence. Therefore, the prediction of supersecondary structure is an important step in the prediction of protein spatial structure. The supersecondary structural motifs are composed of a few secondary structural elements (namely, *α* or *β*) connected by loops. At present, there are four kinds of simple supersecondary structures, namely, *α*-loop-*β*, *α*-loop-*α*, *β*-loop-*α*, and *β*-loop-*β*. These motifs play an important role in protein folding and stability because a large number of motifs exist in protein spatial structure. Many researches have focused on exploring methods for protein supersecondary structure prediction [1, 2]. In 1995, Sun et al. predicted protein supersecondary structure and achieved an accuracy of between 70 and 80% by using neural networks [3]. Chou and Blinn presented a method for predicting beta turns [4–6], alpha turns [7], and all the tight turns [6]. Cruz et al. identified *β*-hairpin and non-*β*-hairpin [8]. Hu and Li identified four kinds of simple supersecondary structures in 2088 proteins and achieved an accuracy of 78~83 % [9]. Zou et al. also predicted four kinds of simple supersecondary structures from 3088 proteins by using support vector machine [10]. And the overall accuracy of 78% was achieved. The features of these studies were mainly derived from the amino acid compositions or dipeptide compositions.

Nuclear magnetic resonance (NMR) technique plays an important role in the determination of three-dimensional biological macromolecule structures. NMR chemical shifts encode subtle information about the local chemical environment of nuclear spins. For many years, there has been growing interest to access this information and utilize it for biomolecular structure determination [11, 12]. Recent progress was made by combining chemical shifts with protein structure prediction programs [13–20], showing that chemical shifts information is a power parameter for the determination of protein structure. In this paper, we utilized chemical shifts as parameters to predict four kinds of simple supersecondary structures in protein by the method of quadratic discriminant analysis. Using the benchmark dataset, we achieved the average of sensitivity of 76.3% and specificity of 74.3% and the overall prediction accuracy of 77.3% in threefold cross-validation by using six CSs (*C*, *C*_*α*_, *C*_*β*_, *H*, *H*_*α*_, *N*) as features. Moreover, we have performed the prediction by combining the different chemical shifts as features. Results showed that the redundant information has great influence on the accuracy.

## 2. Materials and Methods

### 2.1. Database

The chemical shifts of all nuclei (*C*, *C*_*α*_, *C*_*β*_, *H*, *H*_*α*_, *N*) in proteins were extracted from re-referenced protein chemical shift database (namely, RefDB [21]). The following steps were performed to construct the dataset. Firstly, only proteins with six nuclei assigned CSs were considered. Secondly, only proteins with the supersecondary structures information in ArchDB40 [22] were available. We finally utilized the PISCES program [23] to remove the highly similar sequences. After strictly following the aforementioned procedures, 114 proteins were obtained which have both CSs and supersecondary structures. Among 114 proteins, 92% (105 sequences) proteins have less than 25% sequence identity, and the sequence identity of the remains ranges from 25 to 30%. The appendix lists 114 proteins used in this study. Finally, we obtained 90 *α*-loop-*α* (*HH*), 89 *α*-loop-*β* (*HE*), 97 *β*-loop-*α* (*EH*), and 122 *β*-loop-*β* (*EE*) motifs, including the *β*-*β* link and *β*-*β* hairpin.

### 2.2. Feature Parameter

In the four data subsets {*HH*, *HE*, *EH*, *EE*}, we calculated the averaged CSs of six nuclei for a sequence of length *l* using the following formula:

(1) $$\begin{array}{c} t_{i}=\frac{1}{l}\overset{l}{\underset{i=1}{\sum }}\text{C}{\text{S}}_{i}, \end{array}$$

where *i* = *C*, *C*_*α*_, *C*_*β*_, *H*, *H*_*α*_, *N*. Therefore, a sequence can be converted into a six-dimensional vector *R* : {*t*_*i*_}.

### 2.3. Prediction Algorithm

To design an efficient and accurate predicted algorithm the key step is in protein supersecondary structure prediction. The quadratic discriminant analysis [24] is a power algorithm that has been widely applied in genomic and proteomic bioinformatics. Thus, we used it here to perform prediction.

### 2.4. Quadratic Discriminant Analysis (QD)

For a sequence *X* to be classified, we calculated the averaged CSs of six nuclei using (1). So, the sequence is converted into a six-dimensional vector *R* : {*t*_*i*_}:

(2) $$\begin{array}{c} R=\left\{t_{i}\right\}\;\left(i=C,C_{\alpha },C_{\beta },H,H_{\alpha },N\right). \end{array}$$

Here we integrated six-dimensional vector by using quadratic discriminant analysis function. Consider a sequence *X* is classified into four groups (*HH*, *HE*, *EH*, *EE*). The discriminant analysis function between group *i* and group *j* is defined by

(3) $$\begin{array}{c} \xi_{ij}=\ln p\left(\omega_{i}\;|\;X\right)-\ln p\left(\omega_{j}\;|\;X\right). \end{array}$$

According to Bayes' Theorem, we deduce

(4) ξij=ln⁡pipj−δi−δj2−12ln⁡|Σi||Σj|=(ln⁡pi−12δi−12ln⁡|Σi|)   −(ln⁡pj−12δj−12ln⁡|Σj|).

The result can be generalized to* four* groups directly and described as follows.

Set

(5) $$\begin{array}{c} \eta_{v}=\ln p_{v}-\frac{\delta_{v}}{2}-\frac{1}{2}\ln\left|\Sigma_{v}\right|\\ \;\left(v=HH,EH,HE,EE\right), \end{array}$$

where

(6) $$\begin{array}{c} \delta_{v}={\left(R-\mu_{v}\right)}^{T}\Sigma_{v}^{-1}\left(R-\mu_{v}\right), \end{array}$$

where *p*_*v*_ denotes the number of samples in group *v*, *δ*_*v*_ is the square mahalanobis distance between *R* and *μ*_*v*_ with respect to Σ_*v*_ (note: *μ*_*v*_ and |Σ_*v*_| are calculated in training set), and *μ*_*v*_ denotes chemical shift values of six nuclei *R* : {*t*_*i*_} averaged over group *v*; |Σ_*v*_| is the determinant of matrix Σ_*v*_.

The six-dimensional vector *μ*_*v*_ can be written as

(7) $$\begin{array}{c} \mu_{v}^{\left(i\right)}=\frac{1}{p_{v}}\overset{p_{v}}{\underset{i=1}{\sum }}t_{i}, \end{array}$$

where *v* = *HH*, *EH*, *HE*, *EE*; *i* = *C*, *C*_*α*_, *C*_*β*_, *H*_*α*_, *H*,*N*; Σ_*v*_ is the covariance matrix of 6 × 6 dimension, quantifying correlations between the chemical shifts of six nuclei:

(8) $$\begin{array}{c} \Sigma_{v}=\left[\begin{array}{cccc} {\sigma^{v}}_{1,1} & {\sigma^{v}}_{1,2} & \cdots & {\sigma^{v}}_{1,6}\\ {\sigma^{v}}_{2,1\;} & {\sigma^{v}}_{2,2} & \cdots & {\sigma^{v}}_{2,6}\\ \vdots & \vdots & \vdots & \vdots \\ {\sigma^{v}}_{6,1} & {\sigma^{v}}_{6,2} & \cdots & {\sigma^{v}}_{6,6} \end{array}\right], \end{array}$$

where the element

(9) $$\begin{array}{c} \sigma_{i,j}^{v}=\frac{1}{p_{v}}\sum_{}\left(t_{i}-\mu_{v}^{\left(i\right)}\right)\left(t_{j}-\mu_{v}^{\left(j\right)}\right). \end{array}$$

Here *v* = *HH*, *EH*, *HE*, *EE*; *i*, *j* = *C*, *C*_*α*_, *C*_*β*_, *H*_*α*_, *H*, *N*.

From (4) and (5), we have concluded

(10) $$\begin{array}{c} \xi_{ij}=\eta_{i}-\eta_{j}. \end{array}$$

It can be easily proved that *p*(*ω*_*k*_∣*X*) is the maximum of *p*(*ω*_*v*_∣*X*), if *η*_*k*_ is the maximal one in *η*_*v*_ (*v* = *HH*, *EH*, *HE*, *EE*). Then, we predict that *X* belongs to group *k*.

### 2.5. Correction in the Error Allowed Scope

A sequence *X* is predicted for four kinds of supersecondary structures by using (1)~(10). If *η*_*i*_ is the maximal one in *η*_*k*_ (*k* = *HH*, *EH*, *HE*, *EE*), then we predict that *X* belongs to group *i*. However, there are slight differences among *η*_*k*_ (*k* = *HH*, *EH*, *HE*, *EE*). To correct predicted results, we define the coefficient of the error allowed scope as

(11) $$\begin{array}{c} R=\frac{\eta_{\text{corr}}-\eta_{\text{wro}}}{\eta_{\text{corr}}}, \end{array}$$

where *η*_corr_ denotes *X* belonging to itself class*η*, *η*_wro_ denotes *X* being predicted another class *η*. For example, if *X* is the super-secondary structure of *HH*, then *η*_corr_ is *η*_*HH*_ and *η*_wro_ is the maximum among *η*_*EH*_, *η*_*HE*_, *η*_*EE*_.

### 2.6. Performance Evaluation

In statistical prediction, independent dataset test, cross-validation test, and jackknife test can be used to examine a predictor for its effectiveness in practical application. Among the three test methods, the jackknife test is deemed to be the least arbitrary that can always yield a unique result for a given benchmark dataset [25] and has been widely used to examine the performance of various predictors [26–37]. However, in this study we have used the threefold cross-validation to examine the performance of our method; in order to reduce the computational time, we randomly divided the training set into three parts, two of which are for training and the rest for testing. The process is repeated three times. The following three parameters: sensitivity (SN_*i*_), specificity (SP_*i*_), and overall accuracy (*Q*_total_), are used to evaluate the predictive performance of our approach:

(12) $$\begin{array}{c} \text{S}{\text{N}}_{i}=\frac{\text{T}{\text{P}}_{i}}{\text{T}{\text{P}}_{i}+\;\text{F}{\text{N}}_{i}}\times 100\%, \end{array}$$

(13) $$\begin{array}{c} \text{S}{\text{P}}_{i}=\frac{\text{T}{\text{P}}_{i}}{\text{T}{\text{P}}_{i}+\text{F}{\text{P}}_{i}}\times 100\%, \end{array}$$

(14) $$\begin{array}{c} Q_{\text{total}}=\frac{\sum_{i}\text{T}{\text{P}}_{i}}{N}\times 100\%, \end{array}$$

where *i* = *HH*, *HE*, *EH*, *EE* and TP, FN,  TN, and FP denote, respectively, true positives, false positives, true negatives, and false positives. *N* is total number of sequences in four data subsets.

## 3. Results and Discussion

Under the benchmark dataset, we calculated the average chemical shift values using (1). The sequences from four data subsets are converted, respectively, into six-dimensional vectors, which are derived from chemical shift values of six nuclei; then *μ* is also a six-dimensional mean vector, which is calculated in each of the datasets. In the training sets, determinant and inverse matrix of covariance matrix Σ_*v*_ are calculated. Given a sequence of the testing sets, we may calculate *η*_*v*_ by using (4)~(10) and compare the results. Then the class of sequence *X* was determined by the maximum of *η*_*v*_ (*v* = *HH*, *HE*, *EH*, *EE*). Moreover, the coefficient *R* given in (11) is used to correct predicted results. The current study utilized *R* < 0.4. The results of threefold cross-validation are listed in Table 1.

From Table 1, we can see that the averaged sensitivity, specificity, and overall accuracy of four kinds of supersecondary structures are 76.3%, 74.3%, and 77.3%, respectively, indicating that CSs are highly informative with regard to supersecondary structures.

Generally speaking, chemical shift measurements can be incomplete for a multitude of reasons. Often, chemical shifts can only be assigned partially or are missing. To assess the impact of incomplete chemical shift assignments, we performed the prediction by using the combination of the different chemical shifts as features. The results are shown in Table 2.

From Table 2, we found that omission of some CSs can result in radically different accuracy. Theoretically, incomplete chemical shifts provide relatively less information, so the predicted accuracy is also declined. But it actually did not in prediction. We used CSs of *H*,  *H*_*α*_,  *C* as features and achieved the highest accuracy of prediction, indicating that the results are affected by the redundant data. According to the performances, we concluded that CSs of *N*,  *C*_*α*_,  *C*_*β*_ are the most informative features in the prediction of four kinds of protein supersecondary structures. In addition, the information of *C*,  *H*_*α*_,  *N* is commonly provided in protein database; we achieved the prediction accuracy of 79.1% by using CSs of *C*,  *H*_*α*_,  *N* as the only inputs.

To test the method and facilitate comparison with other features, we used amino acid compositions (AAC) as inputs of the method of quadratic discriminant analysis. The compared results are recorded in Table 2. Compared results show that the performances of CSs are superior to that of AAC for supersecondary structures prediction, except* HE* structure (compared with six CSs).

## 4. Conclusions

In this paper, we have introduced a prediction model for supersecondary structures from protein chemical shifts. Our model is both simple and easy to perform. However, owing to the limitation of both information of supersecondary structures and corresponding chemical shifts of six nuclei that should be considered, only 114 proteins have been selected in this study. Based on the benchmark dataset, we investigated the relationship between supersecondary structures and chemical shifts. We achieved the overall accuracy of 77.3% by using six CSs as features and the maximum overall accuracy of 89.2% by using the combination of CSs of *N*,  *C*_*α*_,  *C*_*β*_. Results show that chemical shift is a good parameter for the prediction of four kinds of protein supersecondary structures. In summary, the chemical shifts will become a new parameter in prediction of the protein supersecondary structures in the near future.

## Figures and Tables

**Table 1:** The predicted accuracies by using six CSs as features (3-fold cross-validation).

Class structure	SN (%)	SP (%)	Average SN (%)	Average SP (%)	*Q*_total_ (%)
	*R* < 0.4			
*HH *	73.0	71.0	76.3	74.3	77.3
*EH *	75.8	78.1			
*HE *	69.0	66.7			
*EE *	87.5	81.4			

**Table 2:** Predicted results of different feature combinations (*R* < 0.4).

Feature combinations	*HH *		*EH *		*HE *		*EE *		Average SN (%)	Average SP (%)	*Q*_total_ (%)
	SN (%)	SP (%)	SN (%)	SP (%)	SN (%)	SP (%)	SN (%)	SP (%)			
*C*, *C*_α_, *C*_β_, *H*, *H*_α_	63.3	77.0	84.5	45.6	34.8	100	71.3	77.0	63.4	74.9	64.6
*C*, *C*_α_, *C*_β_, *H*_α_, *N*	90.0	85.3	66.0	97.0	85.4	86.4	93.4	75.5	83.7	86.1	84.2
*C*, *C*_α_, *C*_β_, *N*	55.6	87.7	61.9	80	44.9	93.0	95.1	52.5	64.4	78.3	66.8
*C*_α_, *C*_β_, *N*	90.0	87.1	94.8	83.6	79.8	93.4	91.0	91.7	88.9	89.0	89.2
*C*, *H*_α_, *N*	90.0	73.6	75.3	82.0	79.8	81.6	73.8	80.4	79.7	79.4	79.1
AAC	73.3	73.6	73.0	77.8	72.4	71.3	77.5	75.8	74.1	74.6	75.8

**Table 3:** PDB 114 chains used in this work.

1a6g	1a6j	1a7g	1ail	1akh	1am7	1avs	1b2v
1b56	1bdo	1bed	1bgf	1bja	1by9	1byf	1c44
1cex	1cy5	1dfu	1dhn	1dqe	1dtl	1dyt	1e0c
1edh	1ejf	1ekg	1epf	1ew4	1f2l	1f35	1f3v
1f80	1F8H	1fdq	1ff3	1fil	1g6a	1g6h	1gaw
1gns	1gnu	1go4	1gwy	1gwy	1h4a	1h70	1hcb
1hfc	1hh8	1hrh	1hsl	1huu	1i4f	1ifo	1iho
1iko	1iw0	1iwm	1j1v	1j54	1j7d	1j97	1jr1
1jiw	1jr2	1jl3	1jrl	1jhf	1k82	1l0s	1l1d
1l6x	1lfo	1ljp	1lld	1m1f	1ml4	1mo1	1mxe
1naq	1ng2	1o15	1o5u	1oqr	1osp	1php	1ppf
1pz4	1q4r	1qav	1qfj	1qg7	1qog	1qst	1r5r
1rro	1rsy	1scj	1slm	1snc	1t15	1tkv	1tn3
1tph	1umu	1uoh	1uuh	1uv0	1vap	1vjh	1ycq
1ze3	256b						

## Appendix

See Table 3.

## References

[1] Blundell T, Carney D, Gardner S (1988). Knowledge-based protein modelling and design. *European Journal of Biochemistry*.

[2] Dyson HJ, Wright PE (1993). Peptide conformation and protein folding. *Current Opinion in Structural Biology*.

[3] Sun Z, Rao X, Peng L, Xu D (1997). Prediction of protein supersecondary structures based on the artificial neural network method. *Protein Engineering*.

[4] Chou KC (1997). Prediction of beta-turns in proteins. *Journal of Peptide Research*.

[5] Chou K-C, Blinn JR (1997). Classification and prediction of *β*-turn types. *Journal of Protein Chemistry *.

[6] Chou K-C (2000). Prediction of tight turns and their types in proteins. *Analytical Biochemistry*.

[7] Chou K-C (1997). Prediction and classification of *α*-turn types. *Biopolymers*.

[8] de la Cruz X, Hutchinson EG, Shepherd A, Thornton JM (2002). Toward predicting protein topology: an approach to identifying *β* hairpins. *Proceedings of the National Academy of Sciences of the United States of America*.

[9] Hu XZ, Li QZ (2008). Prediction of the *β*-hairpins in proteins using support vector machine. *Protein Journal*.

[10] Zou DS, He ZS, He JY, Xia Y (2011). Supersecondary structure prediction using Chou's pseudo amino acid composition. *Journal of Computational Chemistry*.

[11] Case DA (1998). The use of chemical shifts and their anisotropies in biomolecular structure determination. *Current Opinion in Structural Biology*.

[12] Wishart DS, Case DA (2001). Use of chemical shifts in macromolecular structure determination. *Methods in Enzymology*.

[13] Cavalli A, Salvatella X, Dobson CM, Vendruscolo M (2007). Protein structure determination from NMR chemical shifts. *Proceedings of the National Academy of Sciences of the United States of America*.

[14] Shen Y, Lange O, Delaglio F (2008). Consistent blind protein structure generation from NMR chemical shift data. *Proceedings of the National Academy of Sciences of the United States of America*.

[15] Lin H, Ding C, Song Q (2012). The prediction of protein structural class using averaged chemical shifts. *Journal of Biomolecular Structure & Dynamics*.

[16] Mechelke M, Habeck M (2013). A probabilistic model for secondary structure prediction from protein chemical shifts. *Proteins*.

[17] Mielke SP, Krishnan VV (2003). Protein structural class identification directly from NMR spectra using averaged chemical shifts. *Bioinformatics*.

[18] Pastore A, Saudek V (1990). The relationship between chemical shift and secondary structure in proteins. *Journal of Magnetic Resonance*.

[19] Wang Y (2004). Secondary structural effects on protein NMR chemical shifts. *Journal of Biomolecular NMR*.

[20] Mao WS, Cong PS, Wang ZH, Lu LJ, Zhu ZL, Li TH (2013). NMRDSP: an accurate prediction of protein shape strings from NMR chemical shifts and sequence data. *PLoS ONE*.

[21] Zhang H, Neal S, Wishart DS (2003). RefDB: a database of uniformly referenced protein chemical shifts. *Journal of Biomolecular NMR*.

[22] Fernandez-Fuentes N, Hermoso A, Espadaler J, Querol E, Aviles FX, Oliva B (2004). Classification of common functional loops of kinase super-families. *Proteins*.

[23] Wang G, Dunbrack RL (2005). PISCES: recent improvements to a PDB sequence culling server. *Nucleic Acids Research*.

[24] Feng Y, Luo L (2008). Use of tetrapeptide signals for protein secondary-structure prediction. *Amino Acids*.

[25] Chou K-C, Shen H-B (2008). Cell-PLoc: a package of Web servers for predicting subcellular localization of proteins in various organisms. *Nature Protocols*.

[26] Chou K-C (2011). Some remarks on protein attribute prediction and pseudo amino acid composition. *Journal of Theoretical Biology*.

[27] Esmaeili M, Mohabatkar H, Mohsenzadeh S (2010). Using the concept of Chou’s pseudo amino acid composition for risk type prediction of human papillomaviruses. *Journal of Theoretical Biology*.

[28] Hayat M, Khan A (2012). Discriminating outer membrane proteins with fuzzy K-nearest neighbor algorithms based on the general form of Chou’s PseAAC. *Protein and Peptide Letters*.

[29] Ding C, Yuan L-F, Guo S-H, Lin H, Chen W (2012). Identification of mycobacterial membrane proteins and their types using over-represented tripeptide compositions. *Journal of Proteomics*.

[30] Chen C, Shen Z-B, Zou X-Y (2012). Dual-layer wavelet SVM for predicting protein structural class via the general form of Chou’s pseudo amino acid composition. *Protein and Peptide Letters*.

[31] Chou K-C, Shen H-B (2010). Plant-mPLoc: a top-down strategy to augment the power for predicting plant protein subcellular localization. *PLoS ONE*.

[32] Chen W, Feng P-M, Lin H, Chou K-C (2013). IRSpot-PseDNC: identify recombination spots with pseudo dinucleotide composition. *Nucleic Acids Research*.

[33] Chen W, Lin H, Feng P-M, Ding C, Zuo Y-C, Chou K-C (2012). iNuc-PhysChem: a sequence-based predictor for identifying nucleosomes via physicochemical properties. *PLoS ONE*.

[34] Lin H, Chen W, Yuan L-F, Li Z-Q, Ding H (2013). Using over-represented tetrapeptides to predict protein submitochondria locations. *Acta Biotheoretica*.

[35] Lin H, Ding C, Yuan L-F (2013). Predicting subchloroplast locations of proteins based on the general form of Chou’s pseudo amino acid composition: approached from optimal tripeptide composition. *International Journal of Biomathematics*.

[36] Lin W-Z, Fang J-A, Xiao X, Chou K-C (2013). ILoc-Animal: a multi-label learning classifier for predicting subcellular localization of animal proteins. *Molecular BioSystems*.

[37] Xiao X, Wang P, Lin W-Z, Jia J-H, Chou K-C (2013). IAMP-2L: a two-level multi-label classifier for identifying antimicrobial peptides and their functional types. *Analytical Biochemistry*.

